# Multiple Thick Nodular Melanoma: Differentiating Multiple Primaries from the Metastasis of a Previous Single Melanoma

**DOI:** 10.4274/balkanmedj.galenos.2019.2019.4.115

**Published:** 2019-10-28

**Authors:** Albert Alhatem, W. Clark Lambert, Robert A. Schwartz, Ravi J. Chokshi

**Affiliations:** 1 Department of Pathology, Immunology and Laboratory Medicine, Rutgers New Jersey Medical School, New Jersey, USA; 2 Department of Dermatology, Rutgers New Jersey Medical School, New Jersey, USA; 3 Department of Surgery, Division of Surgical Oncology, Rutgers Cancer Institute of New Jersey, New Jersey, USA

To the Editor,

The incidence rates of invasive melanoma have been increasing for the past few decades, with an estimated 96480 new cases of melanoma in the United States and 7230 deaths from the disease in 2019 (1).

Nodular melanoma is the second most common type of melanoma after the superficial spreading type. The term “thick melanoma” is reserved for a large malignant melanoma with a Breslow thickness of >4 mm (2). Multiple primary melanomas are clinically defined either by the presence of ≥2 primary melanomas at the time of diagnosis (3) or by the presence of subsequent multiple lesions occurring after the first primary melanoma (4). However, histologically, multiple primary melanomas are defined by the presence of an in-situ component (5). The risk factors for multiple primary melanomas are Caucasian race; male sex; age of >60 years; presence of atypical and/or dysplastic nevi; family history of melanoma or pancreatic, colorectal, or other cancers; and germline mutations, including CDKN2A, CDK4, MC1R, MITF, and PTEN (3,6,7). Here we present a patient with multiple thick melanomas in the lower extremities. To our knowledge, this is the first case with a multiple primary nodular thick melanoma in the lower extremity with Breslow thickness of >15 mm.

A 63-year-old Hispanic woman presented with six months history of an atypical nevus on the right calf, which was pruritic approximately a year and then started to grow approximately four months later, initially slowly and then more rapidly. Over a 1 week period, the tumor started producing a copious purulent liquid and enlarged rapidly, followed by the appearance of two more raised nodules. Two weeks later, 2 of the 3 nodules started draining purulent discharge. Moreover, her mother had colon cancer and her sister had ovarian cancer. On full skin examination, the patient had two ulcerated pigmented nodules measuring 5.5×4.0×3.6 cm^3^ and 4.5×4.0×2.9 cm (Figure 1a, b). Excisional biopsy revealed malignant melanoma in both the lesions (Figure 1c, d), with tumor cells positive for a panel of melanocytic markers, including HMB45 (Figure 1e). Total body positron emission tomography scan did not reveal any metastasis. The patient underwent wide tumor resection and inguinal sentinel lymph node dissection. Histopathologic examination showed melanoma of nodular type, with Breslow thicknesses of 18 and 15 mm, Clark’s level V, lymphovascular invasion (in the distal lesion only), and stage pT4bN0. Molecular testing for BRAF mutation was negative. The patient returned 2 months later with two more nodules in her left leg, which were diagnosed as nodular type melanoma, with Breslow thickness of 4 mm. A transition to metastasis was suspected and immunotherapy with Nivolumab was initiated. Written informed consent was obtained from the patient.

Differentiating multiple primary melanomas from the metastasis of a previous single melanoma is crucial as the staging and management change dramatically (8). Several studies have compared multiple primary melanomas with single primary melanoma, and many significant differences were found between single primary melanoma and subsequent multiple primary melanomas. However, there were no differences between single primary melanoma and the first multiple primary melanomas. The Gene, Environment, Melanoma study group reported a lower mitotic activity; lower tumor thickness and Clark’s level; and more frequent lentigo maligna melanoma subtype, association with dysplastic nevi, and location on the head/neck in subsequent multiple primary melanomas versus single primary melanoma and in subsequent multiple primary melanomas versus the first multiple primary melanomas (9). These results were challenged by others (7), who reported no differences between single primary melanoma and the latest multiple primary melanomas regarding the mitotic activity, tumor thickness, Clark’s level, ulceration, melanoma subtype, and anatomical site. However, they did not evaluate the presence of associated dysplastic nevi, which is the known risk factor for multiple primary melanomas. Moreover, Clark’s thickness is an important predictive factor for multiple primary melanomas and the subsequent appearance of primary melanomas. Furthermore, multiple primary melanomas cohorts have reported that the 2nd and 3rd primary melanomas are prone to occur at the same anatomic location (7). Interestingly, our patient had been diagnosed with an atypical nevus long before she developed melanoma. In addition, her family had a history of colon and ovarian cancers (first-degree relatives), and the histopathologic findings of *in situ* components were suggestive of multiple primary melanomas diagnosis. These results pose a high risk of future development of melanocytic lesions and a need for extensive screening.

## Figures and Tables

**Figure 1:**
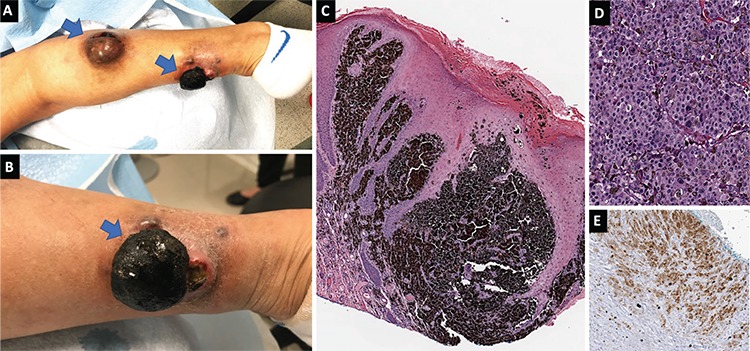
**a-e.** Clinical presentation of multiple primary melanoma in the (a, b) and the histopathologic diagnosis of nodular type (c, d) with positive tumor cells to a panel melanocytic markers, including HMB45 (e).

## References

[1] No authors listed (2019.). American Cancer Society Cancer Facts & Figures 2019 Atlanta:. American Cancer Society.

[2] Ching JA, Gould L (2012). Giant scalp melanoma: a case report and review of the literature. Eplasty.

[3] Cai ED, Swetter SM, Sarin KY (2018.). Association of multiple primary melanomas with malignancy risk: a population-based analysis of the Surveillance, Epidemiology, and End Results Program database from 1973-2014. J Am Acad Dermatol.

[4] Claeson M, Holmström P, Hallberg S, Gillstedt M, Gonzalez H, Wennberg AM, et al (2017). Multiple Primary Melanomas: A Common Occurrence in Western Sweden. Acta Derm Venereol.

[5] Heenan PJ, Ghaznawie M (1999). The pathogenesis of local recurrence of melanoma at the primary excision site. Br J Plast Surg.

[6] Blackwood MA, Holmes R, Synnestvedt M, Young M, George C, Yang H, et al (2002). Multiple primary melanoma revisited. Cancer.

[7] Hwa C, Price LS, Belitskaya-Levy I, Ma MW, Shapiro RL, Berman RS, et al (2012). Single versus multiple primary melanomas: old questions and new answers. Cancer.

[8] Murali R, Scolyer RA, Armstrong BK (2013). Multiple primary cutaneous melanomas: recent studies highlight features associated with more indolent behaviour. Pathology.

[9] Murali R, Goumas C, Kricker A, From L, Busam KJ, Begg CB, et al (2012). Clinicopathologic features of incident and subsequent tumors in patients with multiple primary cutaneous melanomas. Ann Surg Oncol.

